# Assessment of the Impact of Inaccuracy and Variability of Material and Selected Technological Factors on Physical and Mechanical Properties of Fresh Masonry Mortars and Plasters

**DOI:** 10.3390/ma13061382

**Published:** 2020-03-18

**Authors:** Małgorzata Gołaszewska, Jacek Gołaszewski, Grzegorz Cygan, Jerzy Bochen

**Affiliations:** 1 Department of Building Processes Engineering and Building Physics, Faculty of Civil Engineering, Silesian University of Technology, ul. Akademicka 5, 44-100 Gliwice, Poland; jacek.golaszewski@polsl.pl (J.G.); jerzy.bochen@polsl.pl (J.B.); 2 Laboratory of Civil Engineering Faculty, Faculty of Civil Engineering, Silesian University of Technology, ul. Akademicka 5, 44-100 Gliwice, Poland; grzegorz.cygan@polsl.pl

**Keywords:** mortar, lime, variability of technological factors

## Abstract

The article presented the analysis of the impact that various kinds of technological inaccuracies have on the properties of fresh masonry mortars and plasters. Analyzed were the inaccuracies in dosing of mortar components, namely, water, lime, and air-entraining plasticizing admixture (APA) (±10% of mass), and the effect of variable technological conditions, namely, different mixing intensity (fast, slow, normal) and temperature (5 °C, 20 °C, and 35 °C) during first 72 h after mixing. The impact of differences in the properties of cement and aerial (hydrated) lime originating from different manufacturers was also analyzed. The impact of these factors was determined for consistency, density, air content, compressive, and flexural strength. The sensitivity to changes in the analyzed properties was determined by the coefficient of variation. Changes in the dosing of constituents, mixing speed, and temperature adversely affected strength properties. For mortars with APA, these changes exceeded 20% and reached 40%. The greatest impact was evident in the consistency, especially with an excess of APA, where changes ranged from 6% to 80%. The results showed greater resistance of cement-lime mortars to changing selected technological conditions and errors in measuring the amount of ingredients than mortars with air-entraining plasticizing admixture (APA).

## 1. Introduction

Masonry mortars and plasters are the basic types of mortars in traditional building practice, having a significant share in the erection and plastering of masonry walls. Traditionally, masonry mortars are used to connect masonry units, while the plaster is a mortar used to coat interior walls. Apart from function, the main difference between the two types of mortar is the consistency—plasters are more fluid than masonry mortars. Due to the changes in the desired consistency, the composition of the two types of mortar is usually connected to the different water content or admixture content [1]. In addition to system solutions of ready-made mortars, traditional mortars based on individual recipes are still used, including renovation or restoration works, where mortar recipes are often selected individually [2,3,4]. A great deal of attention has been paid to the study of mortar and its properties in the literature, for example, in relation to types of binders, additives, and admixtures, and their impact on physical and mechanical properties. Many mortar tests concern types of aggregate and fillers using waste materials, e.g., concrete waste [5], glass waste [6], brick flour [7], granite flour [8], marble powder [9], and others. A significant part of the research is devoted to modifying mortars with various additives to improve their properties while partially replacing fillers and binders with waste materials.

For example, Harbi et al. [10] carried out the experimental work focused on the study of the possibility of using kaolin dust as filler in the sand, combined with additions of glass powder, brick waste, and metakaolin in order to improve the mechanical performance of the mortar. Mixtures containing glass powder and/or metakaolin turned out to be better than those containing brick waste. Similar research was carried out by Boukour and Benmalek [11], who analyzed the effect of two wastes: crushed clay brick and tire rubber aggregate in cement mortar reinforced by a resinous latex. Test results indicated a decrease in water absorption and shrinkage of such cement mortar.

Atypical addition to mortar, which has been a subject of research, is recycled polyethylene terephthalate (PET) bottles [12]. Test results of this experiment have indicated that higher compressive and flexural strength can be obtained for thusly modified cement mortars. Other atypical additions to the mortar that have been researched are organic agents as vinegar, gram pulse, and frog contaminated water and their influence on mechanical properties and mortar’s performance [13]. It has been concluded that organic materials chosen for the study adversely affect the compressive strength of cement mortars, especially after six months.

Research has also been carried out, attempting to replace cement with other materials in the role of the primary binder. For example, Kim et al. [14] investigated a partial replacement of cement in a mortar with the waste glass sludge. Results showed the strength of such mortars was higher than of mortar with fly ash; however, lower than mortars with ordinary Portland cement only. An interesting study in this area was carried out by Pliya and Cree [15] about adding lime derived from chicken eggshell waste, used as a filler in Portland cement mortar. Although compressive and tensile strengths decreased in comparison to the natural conventional lime mortars, accelerated hydration was gained at the early stages. Jasiczak and Zieliński [16] conducted tests of the effect of protein additives, such as chicken and bovine blood (red cells) on the properties of cement mortar. In a similar vein, Fang et al. [17] and Zhang et al. [18] had recently brought up a topic of properties of traditional Chinese lime mortars with oxblood as an ingredient, which showed that lime mortars with oxblood exhibited better bonding strength and weather resistance (including waterproof quality) than regular lime mortar. The research conducted by Zhao et al. [19] showed that pig blood had a similar beneficial effect on properties, such as early strength.

Modifications of mortar with fibers constitute a separate research group. For example, Çomak et al. [20] studied the effects of hemp fibers on the characteristics of cement-based mortars. Hemp fiber addition had a positive effect on compressive strength, good adherence to cement, and more sufficient bonding between the fibers and mortar matrix. In turn, Trejbal [21] examined the mechanical properties of lime-based mortars reinforced with plasma-treated glass fibers with increased cohesion between their surfaces and lime-based mortar matrix. Attempts were also made to recognize the properties of resin-modified mortars. For example, Schulze, in his studies [22], showed that the addition of a styrene/acrylic polymer binder to cement mortar increased the flexural and peel strength. Moreover, both binders acted in synergy: the cement as the inorganic binder was responsible for mechanical stability as compressive strength, and the styrene/acrylic powder (organic binder) was acting as a reinforcement and was responsible for the internal tensile strength and, at sand-cement interfaces, for the adhesion-bond strength. The properties of mortars with the addition of polyester resin obtained from polyethylene terephthalate (PET) waste bottles have been the subject of other studies [23], which show the beneficial effect of polymers on the physical properties of cement mortars.

There are also known studies on the impact of cement aging on mortar properties [24]. Studies have shown that aging cement negatively impacts mortar flowability, hydration process in mortar during its hardening, and decreases density and compressive strength of hardened mortar. As research has shown, mortar properties are also influenced by factors, such as temperature, dosage method, and curing. For example, the effect of temperature on mortars with the addition of epoxy and polyester resins based on a diglycidyl ether bisphenol A and an aliphatic amine hardener and POLYLITE^®^ 10316, respectively, was studied by Reis [25], who showed a decrease in flexural and compressive strength at higher temperatures. The epoxy mortars turned out to be more sensitive to temperature changes than the polyester ones. Curing conditions, to which a lot of research has been devoted, are important for mortars. Sajedi [26] investigated different methods of curing, including water, air, heated water, oven heated, air-water, and water-air. The highest and lowest compressive strengths were attributed to the mortars with ordinary Portland cement (OPC): water-cured for the duration of 6 h and air-cured in room temperature, oven heated at 60 °C after demoulding of the specimens for 20 h, respectively. In another study, higher strengths have been achieved for ordinary Portland cement and ordinary Portland cement-slag mortars with lower binder content and curing regimen in water without heating [27]. Garijo et al. [28] studied five factors, which had an influence on natural hydraulic lime mortars’ properties, in particular the water binder ratio, the mold material, the aggregate size, and type and curing conditions. The influence of different humidity conditions of curing was tested by Pavlík and Uzáková [29] in their study of the influence of zeolite and metakaolin addition on the compressive strength, porosity, shrinkage, and frost resistance of lime mortars. It was found that pozzolans reduced shrinkage of freshly hydrated lime mortars. These admixtures positively affected compressive strength development and frost resistance of lime-pozzolan mortars when they were cured in the environment with the relative humidity of 100%. As can be seen, the topic of mortars as masonry mortars and plasters is a current one.

Currently, the most common way to improve the properties of masonry mortars and plasters is to use admixtures with different properties depending on their purpose [30,31,32]. These can be admixtures that reduce the amount of water (plasticizing, liquefying), air-entraining, regulating setting and hardening, improving frost-resistance, affecting the water retaining and water resistance, or having a comprehensive effect. The popularization of construction chemicals means that plasticizing admixtures are increasingly used in construction practice instead of the traditional lime [33]. It should be noted that there is a limited amount of research available, which compares the effect of adding plasticizing admixture to cement mortar, to the effect of hydrated lime addition in the binder. The comparison between the effect of lime as a plasticizing agent on mortar properties to an effect of air-entraining plasticizing admixture has been a subject of the paper by Lenart [34]. It was shown that cement-lime mortars were less brittle and had higher compressive and flexural strength than cement mortars with air-entraining and plasticizing admixture, while no unequivocal dependence was confirmed in the case of bond strength of cement-lime mortars and cement mortars with plasticizing admixture. Bond strength of cement-lime mortars has been a subject of research; however, no consensus has been reached, with some authors stating that aerial lime enhances bond strength of cement-lime mortars [35], while others have observed opposite results [36]. Plasticizing admixtures with air-entraining properties, which are popular for masonry mortars and plasters, have been proven to decrease the bond strength [37].

The issue of differences in strength of cement-lime mortars and cement mortar with plasticizing admixture was raised by O’Looney et al. [38]. It was found that depending on the amount of lime, the relation of strength of cement-lime mortars to cement mortars with admixture was different. When lime content was less than 50% of cement mass, the mortars exhibited higher compressive and flexural strength and gained strength at a faster rate than mortars with a plasticizer. However, for mortars with equal amounts of lime and cement, the opposite was true.

Souza et al. [39] made a comparative study of the effect of hydrated lime and three types of additives (air-entraining, plasticizing, and water-retaining) on water retention, density, porosity in a hardened state, and adhesive strength. This research had shown that cement-lime mortars were characterized by the highest density and adhesive strength, as well as the greatest open porosity. Limestone was characterized by the strongest water-retaining characteristics, which was in line with the research of Green et al. [40], whose tests indicated that cement-lime mortars had better water-retaining properties than cement mortars with admixtures. The highest workability was obtained for mortars with water retaining admixture, while the mortars with air-entraining admixture had the lowest open porosity. Addition of plasticizer allowed to obtain average results. It should be noted, however, that the amount of admixtures in the mortar was set to the highest recommended dose, as the composition of mortar was not prepared on the basis of comparable properties (for example, consistency), what is emphasized by the authors.

The research into the effect of proportioning of aerial lime and Portland cement in masonry mortars was conducted by Wright et al. [41]. It was shown that lime mortars had comparable bond strengths but weaker compressive strengths in comparison to cement-lime mortars of the same air content.

The similar effect of aerial lime and plasticizing admixtures on the consistency may, therefore, not provide similarity to the effects on the other properties of plaster and masonry mortar and on the sensitivity of these mortars to dosing errors or varied values of factors affecting fresh mortars, such as temperature, type, and origin of cement or hydrated lime, or mixing intensity [42].

The environmental and technological factors, which may affect the masonry mortar or plaster, have been a subject of numerous research. Lanas et al. [43] investigated mechanical properties of aerial and hydraulic lime-based mortars cured in different environmental conditions and concluded that mechanical properties and durability at different environments were related to the porous structure of mortar. Pavlik and Uzakova [29] tested the humidity effect on shrinkage and compressive strength of aerial lime mortars with the addition of zeolite and metakaolin. It was shown that the mortars with aerial lime and pozzolans had better mechanical properties than aerial lime itself, regardless of humidity. Fusade et al. [44] investigated the difference between the effect of realistic curing conditions to laboratory conditions on the chosen properties of hydraulic lime mortars. Results showed that there were significant differences in the properties of mortars exposed to naturally occurring environmental conditions or laboratory conditions, especially at an early age, mostly due to the assumed high humidity in the realistic curing conditions. Research into the effect of environmental conditions on the properties of fresh and hardened cement mortars are also available in the literature [45,46,47,48].

The influence of the mortar preparation and application process in relation to properties of lime mortars was a subject of research by Cavaco et al. [49], Rosell et al. [50], Balksten et al. [51], and Sandin [52]. It was found that the mechanical properties and consistency of lime mortars could vary depending on the exact preparation process. Davison [53] made studies into the effect of mixing time and procedure on the strength of aerial lime mortars, which implied, that while it is hard to ascertain what is the exact effect of mixing time on properties or mortars, changes in mixing procedure affect the mortar’s strength. The effect of the mixing procedure was also studied by Fukui et al. [54], in the context of cement mortars. It was ascertained that the mixing procedure influenced the amount of air in the case of air-entraining admixture. It should be noted that there is a lack of literature pertaining to the influence of procedures of preparing cement-lime mortar on its properties.

Therefore, to compare the magnitude and significance of changes in material and selected technological factors on the properties of cement-lime mortars without admixtures and mortars with plasticizing admixtures, model tests were conducted in laboratory conditions.

The purpose of the research was to determine and compare the impact of selected material and technological factors on the properties of cement-lime masonry mortars and plasters and cement mortars with an air-entraining plasticizing admixture (APA). The research took into account the impact of cement and hydrated lime origin, inaccuracies in the dosing of water, lime or admixture, as well as the intensity of mortar mixing and temperature changes.

The sensitivity of mortar properties to the impact of the above factors was defined as the coefficient of variation, i.e., the quotient of the absolute measure of the variability of a given property under the influence of a given material or technological factor, and the mean value of this property. The coefficient is a measure of mortar resistance to a change of a specific factor occurring during mortar preparation according to the given recipes.

## 2. Materials and Methods

### 2.1. Preparation of Mortars

For the analysis, six plaster mortars and six masonry mortars with a volume ratio of cement, hydrated lime, and sand of 1:1:6 were prepared. The volume ratio of 1:1:6 was chosen due to the fact that it is one of the traditional but still commonly used volume ratios of cement-lime mortars, and, moreover, it can be effectively used both for masonry mortar and plaster [1]. Ordinary Portland cement CEM I 42.5 R from three different cement plants was adopted for the research. Their chemical composition is shown in Table 1. Mortars were also differentiated by the addition of commercially available hydrated lime from two different producers, marked lime N and lime T. The chemical composition and basic physical properties of the two limes are shown in Table 2. Lime T was characterized by higher Ca(OH)_2_ content; however, lime N had a higher specific surface area.

**Figure 1:**
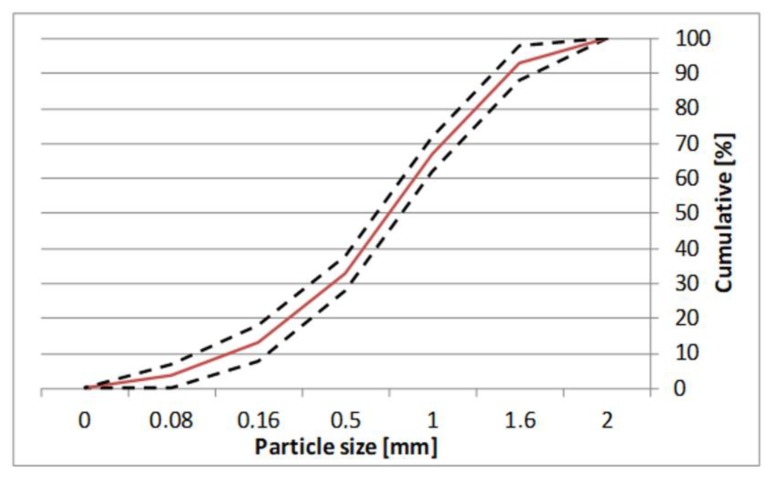
The particle size distribution of standard sand, according to EN-197-1 [55].

To ensure repeatability of the measurement and to minimize the effect of aggregate on the properties of mortars, standard sand 0–2 mm, according to EN-196-1 [55], was used in the research. The particle size distribution of sand used in the research is shown in Figure 1. CEN-Standard sand is an artificially prepared aggregate, consisting of several different sand type fractions, mixed to obtain extremely precise particle size distribution.

The amount of water in the mortar was set to obtain the same consistency, measured by the cone method [56], as it is most often used in building practice. The type of cement significantly affected the amount of water necessary to obtain the required consistency of the tested mortars. The scope of changes in the amount of water in cement-lime and cement mortars with APA was similar. Mortars with lime N were characterized by a slightly higher water-demand than the others. For plaster mortars, a consistency equal to 9 cm by the cone method was adopted, and for masonry—7 cm. The values were chosen on the basis of the traditional consistency of masonry mortars and plaster, according to [1].

In addition, three cement mortars were prepared with a volume ratio of cement to the sand of 1:6 with an APA, acting as “liquid lime”, replacing ordinary hydrated lime, added in an amount of 0.25% of cement mass. This admixture is an aqueous solution of naphthalene resin and surfactants with a density of 1.040 ± 0.03 g/cm^3^, with an alkali content below 5% by mass and chlorides up to 0.1%. In the cement paste, the admixture surrounds the cement grains, giving them a homogeneous charge causing them to repel each other, thus plasticizing the cement mortar, replacing lime as a plasticizing agent. In addition, it improves cohesiveness, prevents segregation, and reduces the surface tension of water, resulting in the formation of stable air micro-pores, which are regularly distributed throughout the mortar volume. The amount of water in these mortars was adjusted to obtain consistency identical to mortars with lime. As a result, nine plasters and masonry mortars were prepared, each with a different composition, as shown in Table 3 and Table 4.

Tests on the variability of technological factors, such as changes in the content of water, lime, or lime-replacing admixtures, as well as types of mixing intensity and temperature deviations, were carried out on mortars with CEM I 42.5 R-C1. Other types of cement were used when testing the effect of cement on the properties of plaster and masonry mortars.

### 2.2. Test Methods

To observe the properties of the analyzed mortars, the following properties were chosen for testing: physical properties, such as consistency, the density of freshly prepared mortar, the air content of the mortar, and flexural and compressive strength. Consistency was determined by three methods: the cone method, the flow table method, and the penetrometer method. Each property was investigated for subsequent dosing mistakes and changes in selected technological factors.

1. The consistency by the cone method was determined on a cone apparatus in accordance with the Polish standard PN-85-B-04500 [56]. The immersion depth is a measure of consistency, which is determined as the mean of three tests.
2. The consistency by the flow table method was determined in accordance with the standard EN 1015-3:2000 [57].
3. The consistency using the plunger penetration apparatus was determined in accordance with the standard EN 1015-4:2000 [58].
4. The fresh mortar density was determined according to the standard EN 1015-6:2000 [59].
5. The air content of the mortar was determined by the pressure method in accordance with EN 1015-7:2000 [60].
6. Tests of the flexural strength and compressive strength were conducted in accordance with the standard EN 1015-11:2001 [61], on a 40 × 40 × 160 mm prismatic specimens. While uniaxial compressive strength test might yield interesting results, as seen in [62,63], the most popular method of strength testing was chosen.

Each tested property was determined for no less than 3 specimens. For compressive strength tests, 40 × 40 × 160 mm prismatic specimens were made according to standard EN 1015-11:2001 [61]. Flexural strength was determined for 3 prismatic specimens, and compressive strength for 6 half-prismatic specimens.

To better present the sensitivity of mortar properties on the impact of the selected factors, a coefficient of variation was calculated. The coefficient of variation is a standard statistical measure of the dispersion of the data obtained. It was calculated as the quotient of the measure of the variability of a given property under the influence of a given material or technological factor and the mean value of this property, as shown in Equation (1).

(1) $$CV=\frac{\sigma }{A}$$

where *CV*—coefficient of variation; *σ*—standard deviation of the results obtained by measuring given property without changes in technological and material factors and with set changes; *A*—arithmetic mean value of all results obtained for a given property (with technological changes and without).

The coefficient can be used as a measure of mortar resistance to a change of a specific factor occurring during mortar preparation, as it accentuates the changes in properties.

## 3. Results and Discussion

Four types of technological factors that could affect selected basic properties of masonry and plaster mortars (namely, bulk density, consistency, air content, flexural, and compressive strength) were adopted for the analysis. Selected technological factors are related to the quantitative and qualitative composition of mortars, taking into account the origin of cement and lime, deviations in the dosing of lime, admixture, and water, as well as changing conditions of mortar preparation usually occurring in construction practice, such as mixing intensity and temperature.

### 3.1. Impact of Cement on the Properties of Plaster and Masonry Mortars

**Figure 2:**
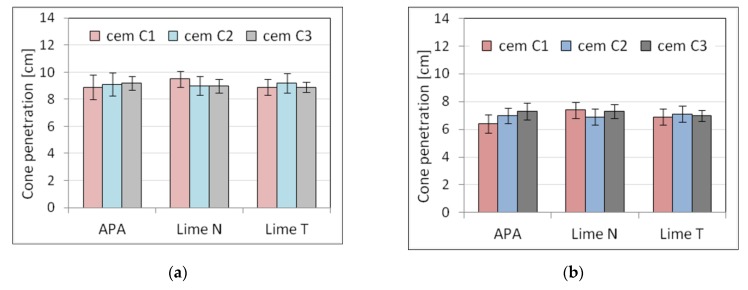
Influence of the cement type on consistency tested by cone method for (**a**) plaster, (**b**) masonry mortars.

**Figure 3:**
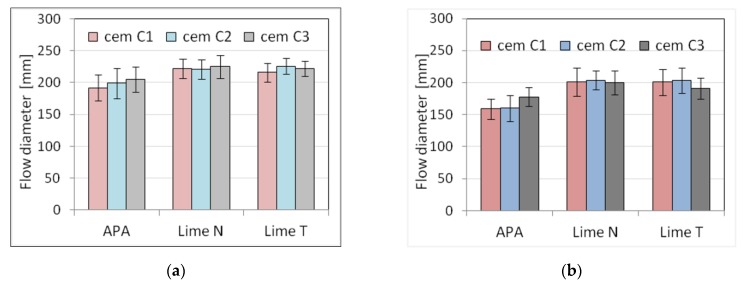
Influence of the cement type on consistency tested by flow table for (**a**) plaster, (**b**) masonry mortars.

**Figure 4:**
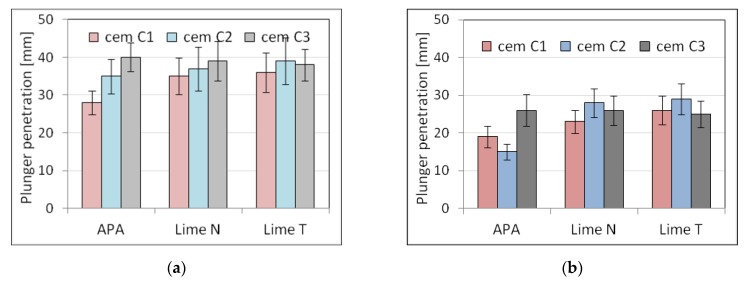
Influence of the cement type on consistency tested by plunger penetration for (**a**) plaster, (**b**) masonry mortars.

**Figure 5:**
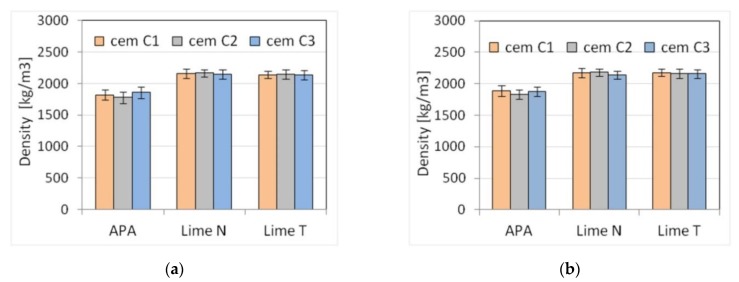
Influence of the cement type on the density of (**a**) plaster, (**b**) masonry mortars.

**Figure 6:**
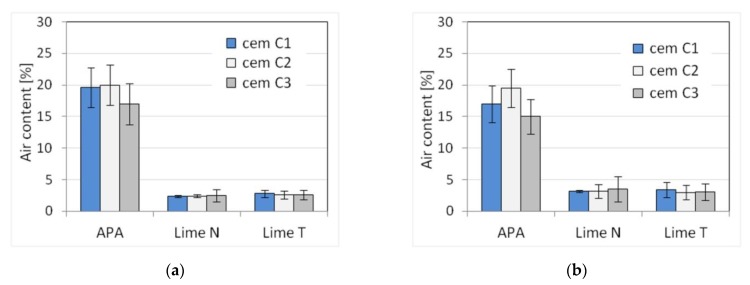
Influence of the cement type on the air content of (**a**) plaster, (**b**) masonry mortars.

**Figure 7:**
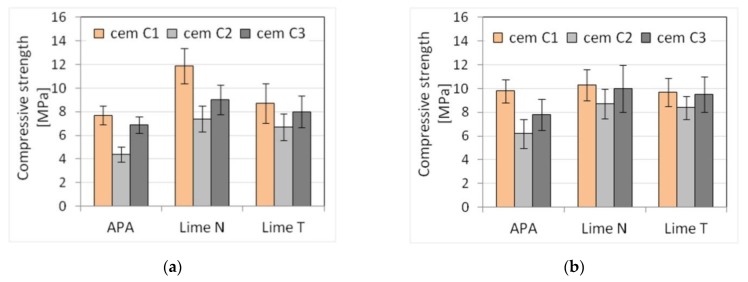
Influence of the cement type on compressive strength of (**a**) plaster, (**b**) masonry mortars.

**Figure 8:**
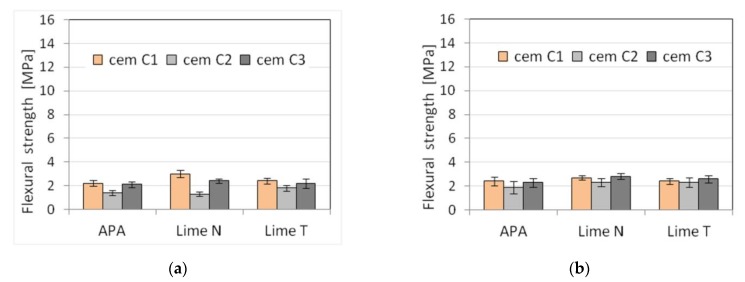
Influence of the cement type on flexural strength of (**a**) plaster, (**b**) masonry mortars.

Five selected physical and mechanical properties were tested on nine types of plasters (Table 3) and nine types of masonry mortars (Table 4). The results of tests of basic properties of the mortars are presented in Table 5 and Table 6 and charts (Figure 2, Figure 3, Figure 4, Figure 5, Figure 6, Figure 7 and Figure 8). Results were the mean of a minimum of three measurements. The standard deviation for each of the results is presented in the corresponding figures.

The consistency results turned out to be diverse in terms of the test method, the origin of cement and lime, and the type of mortar. Due to the fact that the composition of the mortars was set on the basis of the similar consistency measured with the cone method, there was no significant difference between the results of the depth of cone penetration of plasters P1 to P9 and of masonry mortars M1 to M9. However, the differences could be observed in the case of other types of consistency measurement, especially in the case of plunger penetration. This was connected to the fact that the different methods of consistency measurement are not comparable, as they measure the consistency differently in relation to its rheological properties [64]. The greatest difference could be seen in the case of plasters with APA. This effect might be connected to the low viscosity of the mortars with air-entraining admixtures, which could affect the measurement of consistency by penetrometer and flow table [65]. This effect was more visible in the case of plasters due to higher water content. The effect of the APA was connected to the lowering of surface tension of water; therefore, the effect was more pronounced when there was more water in the mortar.

The compressive strength of masonry mortars and plasters with APA is generally lower than in the case of cement-lime mortars. This effect was to be expected due to high air content in those mortars, resulting from using air-entraining admixture [66,67]. The flexural strength, in turn, was less affected by the high air content, which is consistent with the available literature [68,69].

For each tested property, a coefficient of variation was determined, which expressed the sensitivity to changes in the value of a given mortar property under the influence of a change in the amount of ingredients by a specific value. The coefficient was defined as the quotient of the absolute measure of the variability of a given property under the influence of a given material or technological factor, and the mean value of this property (Table 7 and Table 8). The value of the coefficient was determined as a percentage. A higher value means higher susceptibility to changes in properties when the given material or technological factor changes.

Analyzing the results, it could be noticed that the method of consistency testing slightly affected the consistency results. For the cone method of consistency measurement, the values were similar, regardless of the origin of the cement and lime. For the other methods, the addition of lime resulted in more fluid consistency.

Due to set constraints, the masonry mortars obtained a lower consistency than plasters, by about 25% when measured by the cone method. APA as replacement of lime clearly reduced the density by about 15% to a value of the order of magnitude ~1800 kg/m^3^ compared to a density of over 2100 kg/m^3^ for mortars with lime addition (Figure 5). It was expected; as the APA has an air-entraining effect with the increase in air content, the density must decrease. A clear, strong influence of the APA was visible on the air content (Figure 6). Compared to lime mortar, the content increased from 5 to 9 times. In terms of mechanical properties, replacing lime with an APA could cause a reduction in compressive strength of 10–15%. The type of binder did not affect the flexural strength (Figure 7 and Figure 8).

The results showed that the type of cement significantly affected the consistency of mortars and also the amount of water necessary to obtain the required consistency of the tested mortars. The difference in the amount of water differed from 7.2% to 12.5%, the least for lime T, the most for lime N. The scope of this correction was similar in cement-lime mortars and cement mortars with APA. It should be noted that mortars with different types of cement, of equal consistency according to the cone method, were characterized by a different consistency according to the plunger penetration test. This applied especially to cement mortars with APA. The differences in properties of cement of the same class have been thoroughly explored in literature, for example, by Juvas et al. [70], Priyadashana and Dissanayake [71], and, historically, Lyse [72]. This could be explained by the different water demand of types of cement from different manufacturers, which is also one of the reasons why the types of cement used in the tests were characterized, despite the same class, by significant differences in strength. Different water demand for types of cement led to different effective *w*/*c* ratios, and hence to differences in strength. Naturally, the effect was more visible in the case of plasters, which contained more water. This contributed to the increased sensitivity of the properties of mortars to the type of cement and was the reason for a significant difference between the strength of mortars with types of cement from different producers. However, it should be pointed out that the compressive strength of mortars with APA was more sensitive to the type of cement than cement-lime mortars. It must be also noted that with the same consistency, lime mortars had higher compressive strength than cement mortars with APA, which was connected with high air content in mortars with APA.

Analyzing the results of susceptibility to changes of properties due to the type of the binder (Table 7 and Table 8), it could be seen that it was the smallest for the density and consistency determined by the cone method and flow table and amounted to a few percent. Larger changes were visible for consistency using the plunger penetration method, in the order of magnitude of 10%; however, the susceptibility to changes was greater for mortars with APA and amounted up to 10–25%. Higher susceptibility to changes, in the range of 15–35%, was observed for strength properties. The highest variability was noted for plaster mortars, which was caused by its more fluid consistency.

In the case of all tested properties, mortars with APA were characterized by a significantly higher sensitivity to changes in the type of cement than mortars with lime. This might be associated with different compatibility of the admixture with different types of cement; in this case, CEM I 42.5 R cement [30]. However, this effect requires further testing.

### 3.2. Impact of Inaccuracies in Dosing of Lime and Admixture

**Figure 9:**
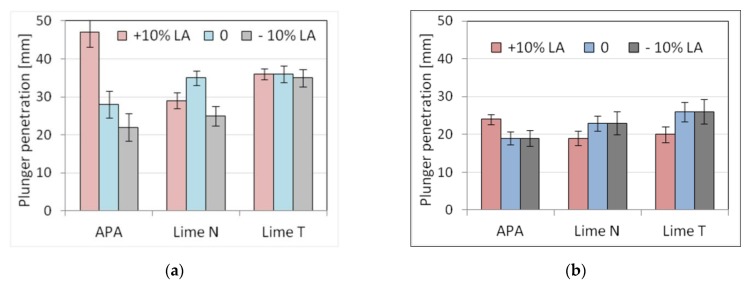
Influence of mistake in dosing lime and admixture on the plunger penetration depth of (**a**) plaster, (**b**) masonry mortars.

**Figure 10:**
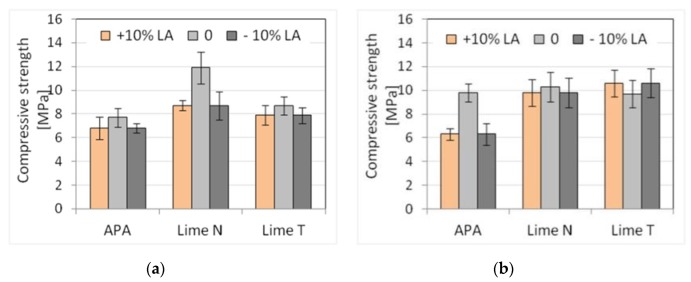
Influence of mistake in dosing lime and admixture on compressive strength of (**a**) plaster, (**b**) masonry mortars.

The tests were performed for three types of plasters and three masonry mortars made with CEM I 42.5 R–C1 cement, different types of lime, and APA. Moreover, the new composition of mortars was made with the addition of lime and APA adjusted by adding 10% more (marked by +10% LA in figures and tables) or 10% less (marked −10% LA) in relation to the initial proportions. This was done to simulate the mistakes in the dosing of lime or admixture that could occur on the construction site. An increase or decrease in the amount of lime or admixture was considered for each mortar. In this way, six types of mortars with three compositions were obtained. For each of the mortars, five properties were determined as previously: consistency, density, air content, flexural, and compressive strength. Results, standard deviation, and the number of samples used are shown in Table 9 and Table 10. The results of the plunger penetration tests are shown in Figure 9, while the compressive strength of mortar is shown in Figure 10, with standard deviation marked for each result.

Analyzing the consistency results, it could be noticed that there were only slight differences in the results for the cone method and the flow table method. Clearer differences were visible for the plunger penetration method, for which less liquid consistencies were obtained for mortars with APA. The differences in the sensitivity of consistency measurements were to be expected, as all three measurements were, in actuality, measurement of slightly different rheological phenomena, which was a subject of the doctoral thesis of Hendrickx [73]. Therefore, the changes in the consistency of mortars for each of the testing methods could be different.

For mortars with the addition of lime, changes in the amount of lime did not affect the consistency, while in the case of using APA, more fluid consistencies were observed than in the case of lime increased by 10% and less liquid when the amount of lime was reduced. The consistency of masonry mortars was, as should be expected, lower than that of plasters, but the differences were dependent on the test procedure. The smallest differences were noticed for the flow table method (around 10%), larger ones (~30%) for the cone method, and the largest (50–70%) for the plunger penetration method (Figure 9). The dosing inaccuracies did not affect the density of the fresh mortar. There was a difference observed between the effect of the APA on mortars in comparison to mortars with the addition of N or T lime, as a decrease in density was observed. The density of mortars with APA addition was around 1850 kg/m^3^, which was about 15% lower in comparison to a density of cement-lime mortars, which was 2150 kg/m^3^. A significant influence of the admixture was visible in the air content, amounting to 17% of air content for masonry mortars and 19% for plasters. Much lower air content could be observed for mortars with lime, in the range of 2–3.7%. However, no clear impact of dosing inaccuracy was observed. In terms of mechanical properties, the effect of dosing inaccuracy was present. Compressive strengths after 28 days reached about 8 MPa for plaster and 10 MPa for masonry mortar (Figure 10). Flexural strengths for all mortars ranged within 2 MPa. A decrease in compressive and flexural strength of 10–20% was observed with both a 10% increase and a decrease in the dosed lime and APA. Major changes occurred in masonry mortars with APA and in plasters with lime N.

The sensitivity of plaster with lime and APA to changes in the amount of lime or admixture (Table 11 and Table 12) was similar in the context of strength, although, in the case of mortars with lime N, the coefficient of variability of strength was clearly higher. In the case of masonry mortars, cement mortars with APA were clearly more sensitive to changes in the amount of admixture in terms of consistency and strength than cement and lime mortars. This might be due to the fact that the absolute amounts of admixture and lime in mortars are of an order of magnitude different so that changes in the content of the admixture would affect the properties of the mortar to a higher degree.

Generally, it can be stated that cement mortars with an APA may show similar or lower resistance to mistakes in dosing of the admixture than cement and lime mortars to mistakes in the dosing of lime.

### 3.3. Impact of Inaccuracies in Dosing Water

**Figure 11:**
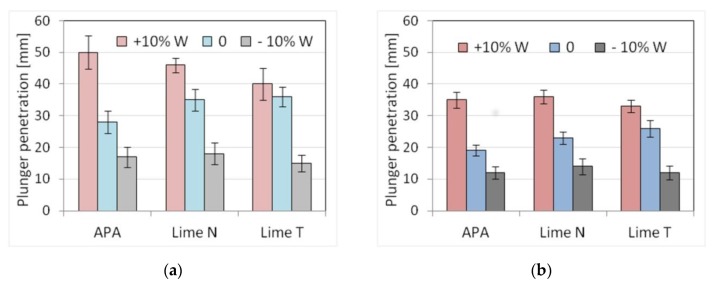
Influence of the errors in water dosing on the consistency tested by plunger penetration method of (**a**) plaster, (**b**) masonry mortars.

**Figure 12:**
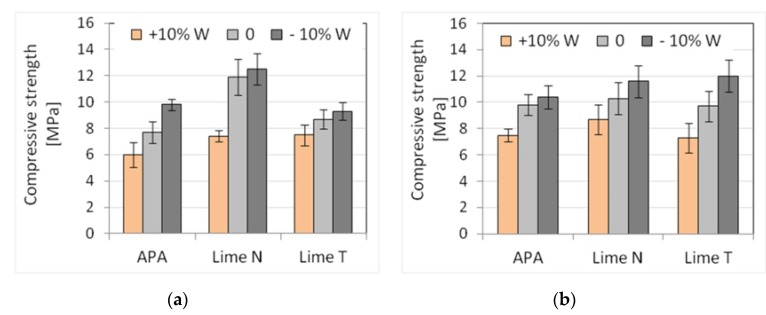
Influence of the errors in water dosing on the compressive strength: (**a**) plaster, (**b**) masonry mortars.

The analysis of the impact of errors in dosing water to the mortar was also carried out for three types of plaster and masonry mortars with cement C1, two types of lime, and the addition of APA. Results, standard deviation, and the number of samples used are shown in Table 13 and Table 14. The results of the plunger penetration tests are shown in Figure 11, while the compressive strength of mortar is shown in Figure 12, with the standard deviation marked for each result. For each mortar, the addition of water was differentiated with an increase or decrease in its amount by 10% compared to the initial proportions (in tables and figures marked +10% W and −10% W respectively). Similarly, as before, five selected physical characteristics were determined for each mortar.

The impact of errors in water dosing was visible in the consistency results. Regardless of the method of measurement, an increase in the amount of water by 10% resulted in greater liquefaction and an increase in the consistency index of about 25–80%, with larger changes occurring when APA was used (Figure 11). In turn, reducing the amount of water by 10% reduced the consistency value in the range of 10–100%. Due to the set conditions, masonry mortars exhibited worse consistencies than plasters, the difference depending on the measurement method. The smallest differences were noted for the flow table method (around 12%), larger ones (~25%) for the cone method, and the largest ones (50%) for the plunger penetration method (Figure 11). For plaster with APA, its density of 1850 kg/m^3^ turned out to be 15% lighter than in the case of plasters with lime (2170 kg/m^3^). Inaccuracies in the dosing of water generally did not affect the density of fresh mortars, although, in the case of masonry mortars with APA, an increase in water by 10% resulted in an additional decrease in density by 25% to a value of 1390 kg/m^3^. Such a significant decrease in density resulted from greater aeration efficiency of the admixture in the presence of more water. In turn, different types of lime did not cause changes in density.

A significant influence of APA was visible for the air content of plasters, in which the amount of air increased six times to the value in the range of 14–21%. In plasters with lime, this content was about 2–4%. In most of the analyzed cases, an increase in the amount of water caused a decrease in air content, while a decrease in the amount of water resulted in its increase. A similar effect was observed by Cesali et al. [74]. On the basis of both research data, it could be concluded that it might be possible that it was due to differences in the volume of porous mortar occupied by water. With more water filling the volume of pores, the volume of free pores decreases, and thus the amount of air contained in them. A different effect occurred in plasters with APA. This might be due to the foaming effect of the admixture in the presence of more water. Water dosing inaccuracy showed its effect on mortar strength.

**Figure 13:**
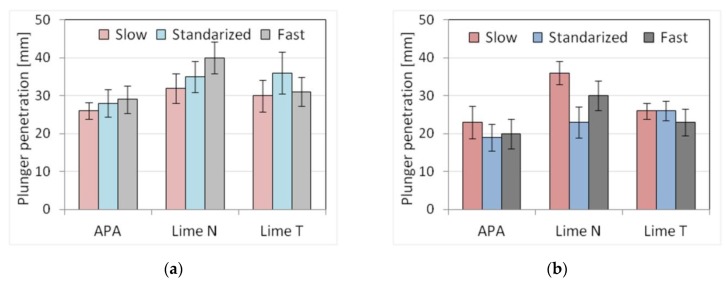
Influence of the mixing intensity on the consistency tested by plunger penetration method of (**a**) plaster, (**b**) masonry mortars.

Generally, the increase in the amount of water caused a decrease in strength in all types of mortars due to the increase of the water-binder ratio. The effect of increased water-binder ratio has been the subject of numerous research, for example, by Elnemr [75], Lawrence [76], and Kim et al. [77]. Both compressive and flexural strength dropped by 12–35%, depending on the type of lime (Figure 13).

The results showed that errors in the dosing of water produced the effects as expected—deterioration of consistency and increase in mortar strength with less water added, and increase in consistency and reduction of mortar strength with more water added. The consistency of mortars changed significantly as a result of changes in the amount of water, and mortars with APA showed greater sensitivity than cement and lime mortars to these changes. Therefore, the sensitivity of consistency, in particular, the consistency determined by the plunger method, to errors in water dosing turned out to be greater compared to other properties (Table 15 and Table 16). This was due to the way the APA works, ensuing high aeration and consistency by reducing the surface tension of the water. Less water made the admixture less effective. The plasticizing effect of lime was associated with the filling effect, as shown by Quadir et al. [78], and this effect was not directly dependent on the water content of the mortar. A smaller amount of air reduced the amount of paste and, in addition to reducing the amount of water, also reduced the consistency. This effect worked in the opposite way in the case of more water, which resulted in increased consistency. Errors in the water dispensing caused significant changes in mortar strength inversely proportional to the amount of water added. However, the sensitivity of compressive strength to errors in the dosing of water for mortars with APA and cement-lime mortars was similar.

### 3.4. Impact of Mixing Intensity

Three plaster and three masonry mortars based on cement C1 with two types of lime and APA were prepared for the test. The results are presented in Table 17 and Table 18.

**Figure 14:**
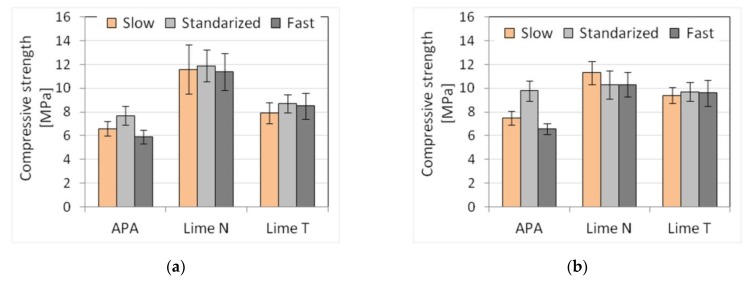
Influence of the mixing intensity on the compressive strength of (**a**) plaster, (**b**) masonry mortars.

The reference mortars were mixed, according to PN-EN 196-1 [56], in a laboratory mixer with rotary and planetary motion, low-speed (140 rpm, 85 rpm) for 2 min and high-speed (285 rpm, 125 rpm) for 1 min. The same mortars were also mixed with two other speeds: intensively mixed with 140 rpm for 3 min (F—fast) and mixed slowly with 90 rpm for 3 min (S—slow). The results of the plunger penetration tests are shown in Figure 13, while the compressive strength of mortar is shown in Figure 14, with standard deviation marked for each result.

Changes in mixing speed had a proportional effect on the consistency of plasters, not exceeding 15%. An increase in speed caused an increase in the consistency and greater fluidity of the plaster. The reverse relationship could be observed in masonry mortars. With regard to consistency, it could be stated that cement-lime mortars were more sensitive to changes in the mixing procedure than mortars with APA (Table 19 and Table 20). This effect might be due to the possibility of uneven distribution of lime in cement-lime mortars, which could occur at high or very low mixing speeds [79]. The admixture was added to the water and mixed by hand, and then added together with water to the mortar, and, therefore, the mixing speed might not be as crucial to an even distribution of the APA in the mortar.

Sensitivity to these changes was highest when determined by the plunger penetration method (Table 19 and Table 20). For masonry mortars, varied changes in consistency were noted due to the changes in mixing speed. Changes in mixing intensity did not affect mortar density or air content. Similar to other analyzed inaccuracies, the replacement of lime with APA caused a decrease in density in the range of 15%. Aerating properties of the admixture resulted in a six-fold increase in air content in comparison to the mortar with lime. The mixing procedure also did not affect the strength of cement-lime mortars, while significantly affecting the strength of cement mortar with APA, up to 35% at higher mixing speed. In this respect, cement mortars with APA showed the greatest sensitivity to changes (Table 19 and Table 20). Interestingly, the standard mixing procedure allowed to obtain cement mortars with APA of the highest strength, while lower or higher intensity caused a decrease in strength. This was despite the fact that the procedure had no effect on the consistency of these mortars, and there was no significant effect on their air content.

### 3.5. Effect of Mortar Temperature

**Figure 15:**
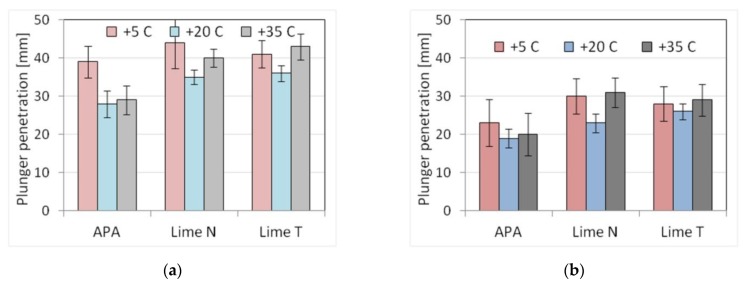
Influence of the temperature changes on the consistency tested by plunger penetration method of (**a**) plaster, (**b**) masonry mortars.

**Figure 16:**
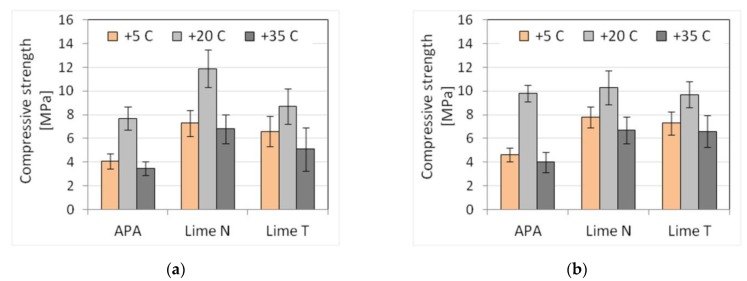
Influence of the temperature changes on the compressive strength of (**a**) plaster, (**b**) masonry mortars.

The last technological factor examined was the temperature and the impact of its change. Three temperatures were analyzed: normal +20 °C, reduced +5 °C, and elevated +35 °C. To obtain the appropriate temperature, the mortar components were properly cooled or heated, so that after mixing, the set temperature was obtained. Only temperatures above zero were taken under consideration due to technical difficulties of obtaining conditions in the laboratory, allowing for keeping the temperature of the fresh mortar below zero. After determining the properties of fresh mortars, samples for strength tests were stored in different ways. Samples of mortars in temperature of +20 °C were cured in accordance with PN-EN 1015-11 [60]. Samples of mortars at +5 °C and +35 °C were stored in the molds for the first 72 h at their original temperature. After demolding, the samples were stored for 25 days at 20 °C and 60% relative humidity. The results are presented in Table 21 and Table 22. The results of the plunger penetration tests are shown in Figure 15, while the compressive strength of mortar is shown in Figure 16, with standard deviation marked for each result.

The temperature of fresh mortars, both reduced and elevated, resulted in an increase in the consistency in the range of 5–26%, i.e., greater mortar liquefaction. Larger changes occurred for the reduced temperature: up to 20% for mortars with the addition of lime, and up to 30% for mortars with APA. The smallest effect was visible for the flow table method, and the largest for the plunger penetration method (Figure 15). The temperature did not affect the mortar density, but due to the APA’s aerating properties, a decrease in density by an average of 16% could be observed in mortars with APA, similar to the previously analyzed cases. The impact of temperature changes on the content of the air was noticeable for masonry mortars. An increase in air content in the range of 2–13% was recorded for both +5 °C and +35 °C. For mortars with the addition of lime, the air content was 2.2–4.9%, and, for mortars with APA, it was 17–19.5%. On the other hand, in the case of mechanical properties, a clearly reducing impact of temperature changes could be observed, resulting in a reduction of strength by 23–59%. Decreasing or increasing the temperature in the examined range of variability reduced the strength of all tested mortars. Larger reductions occurred for mortars with APA (Figure 16).

Plasters with APA showed a higher sensitivity of consistency to temperature changes than cement-lime mortars, while, in the case of masonry mortars, the sensitivity was similar or lower (Table 23 and Table 24). At the same time, in terms of strength, the sensitivity of cement mortars with APA was definitely higher than cement-lime mortars (Table 23 and Table 24). The mechanism of the lower sensitivity of cement-lime mortars could be associated with two effects, connected to the temperature of +5 °C and +35 °C. At +5 °C, the binder, of which there is more in a cement-lime mortar, gave a greater exothermic effect, thus reducing the adverse effect of low temperature. While the hydrated lime used in the research did not react in an exothermic manner, as proven by Cizer et al. [80], and the lime could have a filler effect on cement. Due to its high specific surface area, lime could act as a filler, not allowing for the cement particles to conglomerate, and thus affecting the hydration rate on a physical level [81,82]. At a temperature of +35 °C, lime, as a component with a high ability to bind and hold water, reduced the possibility of rapid loss of water due to which the mortar had better maturing conditions. Those effects were not present in mortars with APA. Moreover, the admixtures might exhibit an erratic or diminished effect on the properties of mortars and concrete, as was proven by Tynes [83] and Silva et al. [84].

### 3.6. General Remarks

The conducted tests and presented relationships indicated greater variability and sensitivity of the properties of cement mortars with APA to technological factors and possible errors in dosing than cement-lime mortars, both in terms of consistency and strength. However, it should be noted that:

- the variability of cement mortars with the analyzed admixture in terms of consistency measured by the cone did not exceed 15%. If expressed in absolute values for plaster and masonry mortars, it translated to ±0.7 and ±0.5 cm, respectively, which was within the permissible error;
- the variability of strength of cement mortar with APA exceeded 20%, while, for cement-lime mortar, it was noticeably lower than 20%; strength of cement-lime mortars was not only more stable but also at the same consistency greater than for cement mortars with APA.

In the case of cement mortars with APA, fluctuations in mortar density were also much larger. Due to the aerating properties, the admixture reduced the mortar density by approximately 15%. The variation of air content in the mortar was smaller for these mortars; however, one should take into account the significant difference in the amount of air, which, in case of cement-lime mortars, was in the range of 2–4%, and, for cement mortars with APA, it was in the range of 17–20%. The variability of the amount of air expressed in absolute values was about 2.5% for cement mortars with APA and about 1% for cement-lime mortars.

The type of cement significantly affected the consistency of mortars. Changing the type of cement assuming a constant mortar consistency necessitated the correction of the amount of water in both cement-lime mortars and cement mortars with APA. In the case of the latter, it could be a correction of the amount of admixture. In the case of cement used in the tests, the amount of water correction for cement-lime mortars and cement mortars with APA was similar and amounted to about 10%. The compressive strength of cement mortars with APA was more sensitive to a change in cement type than cement-lime mortars.

Errors in the dosing of water were particularly evident in the consistency and strength of mortar, resulting in significantly greater changes in percentage than the error value. Water inaccuracy also affected the air content due to the water filling the space occupied by air. Additional water, therefore, reduced the amount of air. This effect was more pronounced in the presence of APA. Changes in strength could be associated with the water-binder ratio.

Changes in the intensity of mixing were unlikely to have a clear impact on the changes in the analyzed mortar properties. They were most visible for the consistency determined by the plunger penetration test, where the increase in mixing speed caused greater liquefaction. In plaster mortars with higher cone or plunger penetration depth, more intensive mixing had a fluidizing effect. In masonry mortars with lower penetration depth, the effect was the opposite. The impact of changes in mixing speed on strength was only visible for cement mortars with APA. Changes in mixing speed reduced the compressive and flexural strength. This was probably related to the plasticizing and aerating effect of the admixture. In this respect, mortars showed the greatest sensitivity to changes.

A similar effect as the one mentioned above occurred when the temperature of the fresh mortar changed. Both decreasing and increasing the temperature of mortar increased its consistency and liquefaction, as well as reduced strength. Lower temperature decreased the hydration and hardening speed of cement paste in mortars. The increased temperature might also affect this process, mostly by causing rapid hydration on the surface of cement particles, which could obstruct the hydration of deeper layers of cement particles [85,86].

## 4. Conclusions

The tests showed greater resistance of cement-lime mortars to changes in the properties of materials, e.g., cement or lime, errors in measuring the amount of ingredients—lime, admixtures, or water, and changing technological conditions, such as mixing intensity, temperature, than mortars with APA.

1. The impact of changing the type of cement was stronger than changes in the type of lime and was most pronounced in strength characteristics. In the case of a synergistic effect or the use of APA, these changes might reach about 50%.
2. Errors in the dosing of lime and admixture, both excess and deficiency, adversely affected strength characteristics. The greatest impact, however, could be noticed in the case of consistency, which changed significantly with an excess of APA. The data, in this case, had a high dispersion, which confirmed the fact that the factor of error in the dosing of constituents of the mortar had a profound effect on the properties, both in case of fresh properties (consistency) and for hardened mortar (strength).
3. Errors in water dosing translated into changes in all the characteristics of fresh mortars, especially the consistency and air content. With excess water, the air content in the mortar decreased, and vice versa. On the other hand, excess water significantly reduced strength, and a deficit of 10% of water contributed to an increase in strength of up to twenty percent.
4. The intensity of mixing directly translated into consistency; however, these changes were more visible, almost proportional, in case of plasters. The opposite relationship prevailed in mortars with lower consistency. The intensity of mixing did not affect the strength of cement-lime mortars, while significantly affecting the strength of cement mortar with an APA admixture, up to 35% at higher mixing speed. The standard mixing procedure allowed to obtain cement mortars with APA of the highest strength, while lower or higher intensity resulted in reduced strength.
5. The reduced and elevated temperature of fresh mortars affected the liquefaction and the consistency for both mortars with lime and APA. Similarly, temperature changes affected the reduction of mortar strength, similar to compression and stretching, with lowering and increasing the temperature.
6. Dosing errors, changes in material properties, and technological factors did not affect the density and air content. These properties were strongly influenced by the APA content in cement mortar.

The tests performed showed that during mortar application, dosing errors and deviations of technological factors had an impact on physical and strength properties. In addition, the possibility of obtaining a mortar that did not meet the requirements when using cement and lime mortars was less than for cement mortars with APA. At the same time, they did not undermine the possibility of effectively using the admixture instead of adding lime to obtain the desired properties of plaster and masonry mortars.

## Figures and Tables

**Table 1:** Chemical composition of types of cement used in the research.

Cement Type	Chemical Composition (% of Weight)											
	CaO	SiO_2_	Al_2_O_3_	Fe_2_O_3_	CaO	MgO	Na_2_0	K_2_O	Na_2_O_eq_	SO_3_	Cl	Insoluble Residue
CEM I 42.5R-C1	20.55	4.67	2.8	64.35	1.18	0.18	0.43	0.46	2.79	0.015	0.6	2.8
CEM I 42.5R-C2	19.9	6.2	2.7	62.6	1.5	0.33	0.72	0.8	2.6	0.05	0.6	2.9
CEM I 42.5R-C3	21.17	3.56	3.23	66.03	0.74	0.19	0.34	0.41	2.55	0.063	0.5	2.2

**Table 2:** Characteristics of hydrated lime N and lime T used in the research.

Properties of Lime		Lime N	Lime T
Chemical composition (% of weight)	CaO + MgO	92.0	96.1
	MgO	1.0	0.7
	CO_2_	2.5	1.0
	SO_3_	0.7	0.1
	Ca(OH)_2_	90.1	92.9
	Unbound water	2.0	0.6
Bulk density (kg/dm^3^)		0.41	0.46
specific surface area by BET method(m^2^/g)		20	19

**Table 3:** The initial composition of plasters (kg/m^3^).

Constituent (kg)	Mortar Type								
	P1	P2	P3	P4	P5	P6	P7	P8	P9
CEM I 42.5 R–C1	169	186	184	-	-	-	-	-	-
CEM I 42.5 R–C2	-	-	-	165	189	186	-	-	-
CEM I 42.5 R–C3	-	-	-	-	-	-	171	184	183
Air entraining Plastizing Admixture (APA)	0.426	-	-	0.426	-	-	0.426	-	-
Lime N	-	89	-	-	91	-	-	89	-
Lime T	-	-	88	-	-	90	-	-	88
Sand	1455	1599	1582	1417	1629	1600	1469	1586	1575
Water	197	284	287	195	260	271	217	287	292

**Table 4:** The initial composition of masonry mortars (kg/m^3^).

Constituent (kg)	Mortar Type								
	M1	M2	M3	M4	M5	M6	M7	M8	M9
CEM I 42.5 R–C1	177	189	189	-	-	-	-	-	-
CEM I 42.5 R–C2	-	-	-	171	192	189	-	-	-
CEM I 42.5 R–C3	-	-	-	-	-	-	174	185	187
Air entraining Plastizing Admixture (APA)	0.446	-	-	0.446	-	-	0.446	-	-
Lime N	-	91	-	-	92	-	-	89	-
Lime T	-	-	91	-	-	91	-	-	90
Sand	1525	1625	1625	1473	1649	1624	1497	1593	1608
Water	189	271	274	188	250	257	204	270	274

**Table 5:** Results of tests of the influence of cement type on properties of plaster.

Property	Statistics	Mortar Type								
		P1	P2	P3	P4	P5	P6	P7	P8	P9
Density (kg/m^3^)	Mean value	1821	2158	2141	1778	2169	2147	1857	2146	2138
	Standard deviation	60.3	90.6	75.3	80.3	72.1	86.2	56.5	86.3	72.1
	No. of samples	6	6	6	6	6	6	6	6	6
Depth of cone penetration (cm)	Mean value	8.9	9.5	8.9	9.1	9	9.2	9.2	9	8.9
	Standard deviation	0.58	0.88	0.6	0.72	0.77	0.85	0.93	0.83	0.73
	No. of samples	6	6	6	6	6	6	6	6	6
Flow diameter (mm)	Mean value	192	222	216	199	221	226	205	225	222
	Standard deviation	15.2	21.1	17.3	17.3	20	20.8	22.3	19.6	16.3
	No. of samples	6	6	6	6	6	6	6	6	6
Depth of plunger penetration (mm)	Mean value	28	35	36	35	37	39	40	39	38
	Standard deviation	3.2	4.8	5.2	4.6	5.8	6.2	3.8	5.2	4.2
	No. of samples	6	6	6	6	6	6	6	6	6
Air content *A_c_* (%)	Mean value	19.6	2.4	2.8	20	2.4	2.6	17	2.5	2.6
	Standard deviation	2.4	0.16	0.18	3.24	0.18	0.2	1.8	0.2	0.23
	No. of samples	6	6	6	6	6	6	6	6	6
Flexural strength (MPa)	Mean value	2.2	3	2.4	1.4	1.3	1.8	2.1	2.4	2.2
	Standard deviation	0.3	0.4	0.21	0.17	0.15	0.19	0.2	0.19	0.25
	No. of samples	3	3	3	3	3	3	3	3	3
Compressive strength (MPa)	Mean value	7.7	11.9	8.7	4.4	7.4	6.7	6.9	9	8
	Standard deviation	0.82	1.67	0.72	0.55	0.88	0.52	0.62	1.1	0.72
	No. of samples	6	6	6	6	6	6	6	6	6

**Table 6:** Results of tests of the influence of cement type on properties of masonry mortars.

Property	Statistics	Mortar Type								
		M1	M2	M3	M4	M5	M6	M7	M8	M9
Density (kg/m^3^)	Mean value	1892	2176	2179	1832	2183	2161	1876	2138	2159
	Standard deviation	70.6	80.3	68.2	59.3	66.4	70.1	67.5	86.2	78.3
	No. of samples	6	6	6	6	6	6	6	6	6
Depth of cone penetration (cm)	Mean value	6.4	7.4	6.9	7	6.9	7.1	7.3	7.3	7
	Standard deviation	0.36	0.68	0.49	0.62	0.52	0.62	0.83	0.8	0.7
	No. of samples	6	6	6	6	6	6	6	6	6
Flow diameter (mm)	Mean value	159	201	201	160	204	203	178	200	191
	Standard deviation	12.1	17.3	18.5	14.2	23.5	20.8	17.2	19.1	17.6
	No. of samples	6	6	6	6	6	6	6	6	6
Depth of plunger penetration (mm)	Mean value	19	23	26	15	28	29	26	26	25
	Standard deviation	2.8	2.1	4.2	3.1	3.8	3.9	3.8	4.1	3.5
	No. of samples	6	6	6	6	6	6	6	6	6
Air content *A_c_* (%)	Mean value	17	3.2	3.4	19.5	3.2	3	15	3.5	3.1
	Standard deviation	1.52	0.23	0.26	2.32	0.27	0.23	1.28	0.3	0.26
	No. of samples	6	6	6	6	6	6	6	6	6
Flexural strength (MPa)	Mean value	2.4	2.7	2.4	1.9	2.3	2.3	2.3	2.8	2.6
	Standard deviation	0.28	0.24	0.19	0.17	0.34	0.25	0.32	0.51	0.31
	No. of samples	3	3	3	3	3	3	3	3	3
Compressive strength (MPa)	Mean value	9.8	10.3	9.7	6.2	8.7	8.4	7.8	10	9.5
	Standard deviation	1.22	1.34	1.11	0.7	0.66	0.68	0.88	1.2	0.92
	No. of samples	6	6	6	6	6	6	6	6	6

**Table 7:** The sensitivity of properties of plaster to changing cement type.

Property	APA	Lime N	Lime T
Sensitivity–coefficient of variation (%)			
Density	2.2	0.5	0.2
Depth of cone penetration	1.7	3.1	1.9
Flow diameter	3.3	0.9	2.3
Depth of plunger penetration	17.6	5.4	4.1
Air content *A_c_*	8.6	2.4	4.3
Compressive strength	27.2	24.2	13.0
Flexural strength	22.9	38.6	14.3

**Table 8:** The sensitivity of properties of masonry mortar to changing cement type.

Property	APA	Lime N	Lime T
Sensitivity–coefficient of variation (%)			
Density	1.7	1.1	0.5
Depth of cone penetration	6.6	3.7	1.4
Flow diameter	6.5	1.0	3.2
Depth of plunger penetration	27.8	9.8	7.8
Air content *A_c_*	13.1	5.2	6.6
Compressive strength	22.7	8.8	7.6
Flexural strength	12.0	10.2	6.3

**Table 9:** Results of tests of the influence of mistakes in dosing of lime/APA on properties of plaster.

Property	Statistics	Reference			+10% LA			−10% LA		
		P1	P2	P3	P1	P2	P3	P1	P2	P3
Density (kg/m^3^)	Mean value	1821	2158	2141	1783	2151	2166	1833	2160	2161
	Standard deviation	60.3	90.6	75.3	50.5	39.8	81.4	92.1	80.4	81.2
	No. of samples	6	6	6	6	6	6	6	6	6
Depth of cone penetration (cm)	Mean value	8.9	9.5	8.9	9.1	9.5	8.9	8.2	9.1	9.4
	Standard deviation	0.58	0.88	0.6	0.51	0.61	0.71	0.64	0.71	0.75
	No. of samples	6	6	6	6	6	6	6	6	6
Flow diameter (mm)	Mean value	192	222	216	200	223	221.5	185.5	227.5	226
	Standard deviation	15.2	21.1	17.3	15.2	19.2	20	15.2	17.8	18.1
	No. of samples	6	6	6	6	6	6	6	6	6
Depth of plunger penetration (mm)	Mean value	28	35	36	47	29	36	22	25	35
	Standard deviation	3.2	4.8	5.2	3.6	2.6	4.1	2.6	2.7	2.9
	No. of samples	6	6	6	6	6	6	6	6	6
Air content *A_c_* (%)	Mean value	19.6	2.4	2.8	19	2.3	2	18.5	3.3	2.8
	Standard deviation	2.4	0.16	0.18	0.8	0.12	0.14	1.2	0.15	0.14
	No. of samples	6	6	6	6	6	6	6	6	6
Flexural strength (MPa)	Mean value	2.2	3	2.4	2	2.1	2.2	2	2.1	2.2
	Standard deviation	0.3	0.4	0.21	0.12	0.2	0.15	0.1	0.14	0.11
	No. of samples	3	3	3	3	3	3	3	3	3
Compressive strength (MPa)	Mean value	7.7	11.9	8.7	6.8	8.7	7.9	6.8	8.7	7.9
	Standard deviation	0.82	1.67	0.72	0.53	0.92	0.66	0.56	0.68	0.57
	No. of samples	6	6	6	6	6	6	6	6	6

**Table 10:** Results of tests of the influence of mistakes in dosing of lime/APA on properties of masonry mortars.

Property	Statistics	Reference			+10% LA			−10% LA		
		M1	M2	M3	M1	M2	M3	M1	M2	M3
Density (kg/m^3^)	Mean value	1892	2176	2179	1837	2150	2177	1908	2170	2154
	Standard deviation	70.6	80.3	68.2	70.5	65.2	71.2	61.5	71.1	68.9
	No. of samples	6	6	6	4	4	4	4	4	4
Depth of cone penetration (cm)	Mean value	6.4	7.4	6.9	7	7	6.6	5.9	7.5	7.2
	Standard deviation	0.36	0.68	0.49	0.35	0.39	0.49	0.49	0.64	0.35
	No. of samples	6	6	6	6	6	6	6	6	6
Flow diameter (mm)	Mean value	159	201	201	178	202	198.5	145	203.5	206.5
	Standard deviation	12.1	17.3	18.5	17.6	16.5	17.8	16.8	18.1	17.9
	No. of samples	6	6	6	6	6	6	6	6	6
Depth of plunger penetration (mm)	Mean value	19	23	26	24	19	20	17	27	28
	Standard deviation	2.8	2.1	4.2	2.8	1.9	1.65	1.53	1.92	2.22
	No. of samples	6	6	6	6	6	6	6	6	6
Air content *A_c_* (%)	Mean value	17	3.2	3.4	19.8	3.7	2.8	16	3.7	3.4
	Standard deviation	1.52	0.23	0.26	1.8	0.21	0.14	1.1	0.19	0.17
	No. of samples	6	6	6	6	6	6	6	6	6
Flexural strength (MPa)	Mean value	2.4	2.7	2.4	1.9	2.5	2.5	1.9	2.5	2.5
	Standard deviation	0.28	0.24	0.19	0.14	0.2	0.2	0.13	0.19	0.12
	No. of samples	3	3	3	3	3	3	3	3	3
Compressive strength (MPa)	Mean value	9.8	10.3	9.7	6.3	9.8	10.6	6.3	9.8	10.6
	Standard deviation	1.22	1.34	1.11	0.48	0.78	0.88	0.42	0.67	1.02
	No. of samples	6	6	6	6	6	6	6	6	6

**Table 11:** The sensitivity of plaster properties to mistakes in dosing lime or APA.

Property	APA	Lime N	Lime T
Sensitivity–coefficient of variation (%)			
Density	1.4	0.2	0.6
Depth of cone penetration	5.4	2.5	3.2
Flow diameter	3.6	1.4	2.3
Depth of plunger penetration	40.4	17.0	1.6
Air content *A_c_*	1.3	20.7	18.2
Compressive strength	7.3	18.9	5.7
Flexural strength	5.6	21.7	5.1

**Table 12:** The sensitivity of masonry mortars properties to mistakes in dosing lime or APA.

Property	APA	Lime N	Lime T
Sensitivity–coefficient of variation (%)			
Density	1.2	0.6	0.6
Depth of cone penetration	8.6	3.6	4.3
Flow diameter	10.3	0.8	2.1
Depth of plunger penetration	18.0	17.4	16.9
Air content *A_c_*	2.8	8.2	10.8
Compressive strength	27.1	2.9	5.0
Flexural strength	14.0	4.5	2.3

**Table 13:** Results of tests of the influence of water dosing mistakes on properties of plaster.

Propert	Statistics	Reference			+10% W			−10% W		
		P1	P2	P3	P1	P2	P3	P1	P2	P3
Density (kg/m^3^)	Mean value	1821	2158	2141	1816	2137	2174	1900	2155	2171
	Standard deviation	60.3	90.6	75.3	61.2	81.9	82.3	76.5	70.2	82.1
	No. of samples	6	6	6	6	6	6	6	6	6
Depth of cone penetration (cm)	Mean value	8.9	9.5	8.9	12.2	11.2	11.1	6	6.8	6.7
	Standard deviation	0.58	0.88	0.6	0.7	0.71	0.79	0.57	0.54	0.49
	No. of samples	6	6	6	6	6	6	6	6	6
Flow diameter (mm)	Mean value	192	222	216	261.5	251	230	164	196	196.5
	Standard deviation	15.2	21.1	17.3	17.9	18.9	19.1	15.6	17.9	16.3
	No. of samples	6	6	6	6	6	6	6	6	6
Depth of plunger penetration (mm)	Mean value	28	35	36	50	46	40	17	18	15
	Standard deviation	3.2	4.8	5.2	4.2	4.8	5.33	1.9	2.2	2.1
	No. of samples	6	6	6	6	6	6	6	6	6
Air content *A_c_* (%)	Mean value	19.6	2.4	2.8	21	1.2	2.5	15.5	4.2	3.3
	Standard deviation	2.4	0.16	0.18	2	0.11	0.09	1.14	0.31	0.15
	No. of samples	6	6	6	6	6	6	6	6	6
Flexural strength (MPa)	Mean value	2.2	3	2.4	2.1	2.1	2.1	2.4	2.7	2.4
	Standard deviation	0.3	0.4	0.21	0.2	0.19	0.2	0.21	0.23	0.19
	No. of samples	3	3	3	3	3	3	3	3	3
Compressive strength (MPa)	Mean value	7.7	11.9	8.7	6	7.4	7.5	9.8	12.5	9.3
	Standard deviation	0.82	1.67	0.72	0.82	0.88	0.62	1.21	1.75	1.32
	No. of samples	6	6	6	6	6	6	6	6	6

**Table 14:** Results of tests of the influence of water dosing mistakes on properties of masonry mortars.

Property	Statistics	Reference			+10% W			−10% W		
		M1	M2	M3	M1	M2	M3	M1	M2	M3
Density (kg/m^3^)	Mean value	1892	2176	2179	1851	2166	2157	1893	2151	2193
	Standard deviation	70.6	80.3	68.2	75.9	80.1	74.3	78.5	62.3	81.3
	No. of samples	6	6	6	6	6	6	6	6	6
Depth of cone penetration (cm)	Mean value	6.4	7.4	6.9	9.7	10.5	9.3	4.9	5.1	4.8
	Standard deviation	0.36	0.68	0.49	0.87	0.75	0.81	0.36	0.41	0.35
	No. of samples	6	6	6	6	6	6	6	6	6
Flow diameter (mm)	Mean value	159	201	201	210	239	236.5	143.5	172.5	176.5
	Standard deviation	12.1	17.3	18.5	19.5	21.3	18.7	13.2	16.8	14.9
	No. of samples	6	6	6	6	6	6	6	6	6
Depth of plunger penetration (mm)	Mean value	19	23	26	35	36	33	12	14	12
	Standard deviation	2.8	2.1	4.2	4.2	3.1	3.5	1.7	1.71	2.1
	No. of samples	6	6	6	6	6	6	6	6	6
Air content *A_c_* (%)	Mean value	17	3.2	3.4	19.2	2	2.1	14	4.5	3.6
	Standard deviation	1.52	0.23	0.26	1.87	0.15	0.19	1.24	0.35	0.21
	No. of samples	6	6	6	6	6	6	6	6	6
Flexural strength (MPa)	Mean value	2.4	2.7	2.4	2.1	2.3	2.2	2.4	3.1	2.6
	Standard deviation	0.28	0.24	0.19	0.19	0.18	0.2	0.21	0.29	0.21
	No. of samples	3	3	3	3	3	3	3	3	3
Compressive strength (MPa)	Mean value	9.8	10.3	9.7	7.5	8.7	7.3	10.4	11.6	12
	Standard deviation	1.22	1.34	1.11	0.8	0.92	0.7	1.22	1.23	1.1
	No. of samples	6	6	6	6	6	6	6	6	6

**Table 15:** The sensitivity of plaster properties to mistakes in dosing water.

Property	APA	Lime N	Lime T
Sensitivity–coefficient of variation (%)			
Density	2.6	0.5	0.8
Depth of cone penetration	34.3	24.2	24.7
Flow diameter	24.5	12.3	7.7
Depth of plunger penetration	53.1	42.7	44.3
Air content *A_c_*	15.0	58.1	14.1
Compressive strength	24.3	26.3	10.8
Flexural strength	6.8	17.6	7.5

**Table 16:** The sensitivity of masonry mortars properties to mistakes in dosing water.

Property	APA	Lime N	Lime T
Sensitivity–coefficient of variation (%)			
Density	16.9	0.6	0.8
Depth of cone penetration	35.1	35.3	32.2
Flow diameter	20.2	16.2	14.7
Depth of plunger penetration	53.6	45.5	45.2
Air content *A_c_*	15.6	38.7	26.9
Compressive strength	17.9	14.2	24.3
Flexural strength	6.7	14.8	8.3

**Table 17:** Results of tests of the influence of mixing intensity on properties of plaster.

Property	Statistics	Reference			Fast			Slow		
		P1	P2	P3	P1	P2	P3	P1	P2	P3
Density (kg/m^3^)	Mean value	1821	2158	2141	1823	2164	2162	1846	2157	2133
	Standard deviation	60.3	90.6	75.3	75.1	74.1	90.3	83.6	87.6	79.3
	No. of samples	6	6	6	5	5	5	5	5	5
Depth of cone penetration (cm)	Mean value	8.9	9.5	8.9	9	10.3	8.9	9	8.8	9.3
	Standard deviation	0.58	0.88	0.6	0.7	0.65	0.56	0.83	0.74	0.81
	No. of samples	6	6	6	6	6	6	6	6	6
Flow diameter (mm)	Mean value	192	222	216	204.5	227.5	215.5	196	222.5	226.5
	Standard deviation	15.2	21.1	17.3	18.2	19.3	18.6	17.5	19.1	17.6
	No. of samples	6	6	6	6	6	6	6	6	6
Depth of plunger penetration (mm)	Mean value	28	35	36	29	40	31	26	32	30
	Standard deviation	3.2	4.8	5.2	3	5.48	3.8	3.2	3.9	2.8
	No. of samples	6	6	6	6	6	6	6	6	6
Air content *A_c_* (%)	Mean value	19.6	2.4	2.8	18	2	3	19.5	2.3	3.6
	Standard deviation	2.4	0.16	0.18	1.1	0.1	0.2	1.21	0.12	0.21
	No. of samples	6	6	6	6	6	6	6	6	6
Flexural strength (MPa)	Mean value	2.2	3	2.4	1.6	2.8	2.1	2.3	2.8	1.8
	Standard deviation	0.3	0.4	0.21	0.14	0.19	0.15	0.18	0.21	0.15
	No. of samples	3	3	3	3	3	3	3	3	3
Compressive strength (MPa)	Mean value	7.7	11.9	8.7	5.9	11.4	8.5	6.6	11.6	7.9
	Standard deviation	0.82	1.67	0.72	0.7	1.12	1.09	0.78	1.8	0.89
	No. of samples	6	6	6	6	6	6	6	6	6

**Table 18:** Results of tests of the influence of mixing intensity on properties of masonry mortars.

Property	Statistics	Reference			Fast			Slow		
		M1	M2	M3	M1	M2	M3	M1	M2	M3
Density (kg/m^3^)	Mean value	1892	2176	2179	1845	2173	2157	1830	2162	2141
	Standard deviation	70.6	80.3	68.2	91.2	78.4	72.3	81.1	73.4	89.1
	No. of samples	6	6	6	6	5	5	5	5	5
Depth of cone penetration (cm)	Mean value	6.4	7.4	6.9	6.5	9.9	7.2	6.5	8.1	8
	Standard deviation	0.36	0.68	0.49	0.58	0.87	0.65	0.41	0.45	0.24
	No. of samples	6	6	6	6	6	6	6	6	6
Flow diameter (mm)	Mean value	159	201	201	180	201	197.5	177	211.5	206.5
	Standard deviation	12.1	17.3	18.5	17.1	16.4	13.1	16.9	12.3	14.6
	No. of samples	6	6	6	6	6	6	6	6	6
Depth of plunger penetration (mm)	Mean value	19	23	26	20	30	23	23	36	26
	Standard deviation	2.8	2.1	4.2	2.4	4	2.16	2.9	5.28	3
	No. of samples	6	6	6	6	6	6	6	6	6
Air content *A_c_* (%)	Mean value	17	3.2	3.4	17	3.1	3.8	19	3.3	3.9
	Standard deviation	1.52	0.23	0.26	1.23	0.25	0.31	1.47	0.14	0.21
	No. of samples	6	6	6	6	6	6	6	6	6
Flexural strength (MPa)	Mean value	2.4	2.7	2.4	2	2.6	2.5	2.1	2.4	2.3
	Standard deviation	0.28	0.24	0.19	0.09	0.14	0.14	0.2	0.19	0.19
	No. of samples	3	3	3	3	3	3	3	3	3
Compressive strength (MPa)	Mean value	9.8	10.3	9.7	6.6	10.3	9.6	7.5	11.3	9.4
	Standard deviation	1.22	1.34	1.11	0.62	1.45	2.1	0.56	1.43	0.65
	No. of samples	6	6	6	6	6	6	6	6	6

**Table 19:** The sensitivity of plaster properties to changes in mixing intensity.

Property	APA	Lime N	Lime T
Sensitivity–coefficient of variation (%)			
Density	0.8	0.2	0.7
Depth of cone penetration	0.6	7.9	2.6
Flow diameter	3.4	1.4	2.9
Depth of plunger penetration	5.5	11.3	9.9
Air content *A_c_*	4.0	9.3	13.3
Compressive strength	13.5	2.2	5.0
Flexural strength	18.6	4.0	14.3

**Table 20:** The sensitivity of masonry mortar properties to changes in mixing intensity.

Property	APA	Lime N	Lime T
Sensitivity–coefficient of variation (%)			
Density	1.7	0.3	0.9
Depth of cone penetration	0.9	15.2	7.7
Flow diameter	6.6	3.1	2.3
Depth of plunger penetration	10.1	21.9	6.9
Air content *A_c_*	6.5	3.1	7.2
Compressive strength	20.7	5.4	1.6
Flexural strength	9.6	6.0	4.2

**Table 21:** Results of tests of the influence of temperature on properties of plaster.

Property	Statistics	Reference			+5 °C			+35 °C		
		P1	P2	P3	P1	P2	P3	P1	P2	P3
Density (kg/m^3^)	Mean value	1821	2158	2141	1802	2133	2167	1780	2133	2135
	Standard deviation	60.3	90.6	75.3	80.1	95.3	81.2	71.3	83.2	58.9
	No. of samples	6	6	6	6	6	6	6	6	6
Depth of cone penetration (cm)	Mean value	8.9	9.5	8.9	9.9	10.5	9.9	8.3	9.6	10
	Standard deviation	0.58	0.88	0.6	0.7	0.91	0.75	0.65	0.78	0.91
	No. of samples	6	6	6	6	6	6	6	6	6
Flow diameter (mm)	Mean value	192	222	216	197.5	227	212.5	182	219	226
	Standard deviation	15.2	21.1	17.3	15.5	21.3	15.6	17.2	18.3	16.3
	No. of samples	6	6	6	6	6	6	6	6	6
Depth of plunger penetration (mm)	Mean value	28	35	36	39	44	41	29	40	43
	Standard deviation	3.2	4.8	5.2	6.76	2.4	3.6	3.8	3.2	3.18
	No. of samples	6	6	6	6	6	6	6	6	6
Air content *A_c_* (%)	Mean value	19.6	2.4	2.8	19.4	2.2	3	19	2.5	4
	Standard deviation	2.4	0.16	0.18	0.42	0.12	0.21	0.34	0.15	0.26
	No. of samples	6	6	6	6	6	6	6	6	6
Flexural strength (MPa)	Mean value	2.2	3	2.4	1.3	1.8	2	1.1	1.6	1.4
	Standard deviation	0.3	0.4	0.21	0.12	0.16	0.19	0.1	0.15	0.11
	No. of samples	3	3	3	3	3	3	3	3	3
Compressive strength (MPa)	Mean value	7.7	11.9	8.7	4.1	7.3	6.6	3.5	6.8	5.1
	Standard deviation	0.82	1.67	0.72	0.65	1.1	0.72	0.58	0.88	0.72
	No. of samples	6	6	6	6	6	6	6	6	6

**Table 22:** Results of tests of the influence of temperature on properties of masonry mortars.

Property	Statistics	Reference			+5 °C			+35 °C		
		M1	M2	M3	M1	M2	M3	M1	M2	M3
Density (kg/m^3^)	Mean value	1892	2176	2179	1820	2125	2175	1837	2145	2134
	Standard deviation	70.6	80.3	68.2	72.2	59.6	87.6	91.2	55.5	68.9
	No. of samples	6	6	6	6	6	6	6	6	6
Depth of cone penetration (cm)	Mean value	6.4	7.4	6.9	7.3	9.6	7.6	7	8.1	7.9
	Standard deviation	0.36	0.68	0.49	0.58	0.74	0.25	0.23	0.67	0.57
	No. of samples	6	6	6	6	6	6	6	6	6
Flow diameter (mm)	Mean value	159	201	201	181	218	209	173	210	205.5
	Standard deviation	12.1	17.3	18.5	17.1	14.5	15.1	17.1	20.5	19.8
	No. of samples	6	6	6	6	6	6	6	6	6
Depth of plunger penetration (mm)	Mean value	19	23	26	23	30	28	20	31	29
	Standard deviation	2.8	2.1	4.2	3.2	4.22	2.6	1.62	3.9	3.85
	No. of samples	6	6	6	6	6	6	6	6	6
Air content *A_c_* (%)	Mean value	17	3.2	3.4	19.5	4.9	3.7	18.5	4.2	4.4
	Standard deviation	1.52	0.23	0.26	1.24	0.47	0.14	1.51	0.21	0.32
	No. of samples	6	6	6	6	6	6	6	6	6
Flexural strength (MPa)	Mean value	2.4	2.7	2.4	1.4	1.9	2.2	1.1	1.8	1.7
	Standard deviation	0.28	0.24	0.19	0.08	0.19	0.18	0.1	0.17	0.14
	No. of samples	3	3	3	3	3	3	3	3	3
Compressive strength (MPa)	Mean value	9.8	10.3	9.7	4.6	7.8	7.3	4	6.7	6.6
	Standard deviation	1.22	1.34	1.11	0.62	1.14	0.98	0.71	0.83	0.58
	No. of samples	6	6	6	6	6	6	6	6	6

**Table 23:** The sensitivity of plaster properties to temperature changes.

Property	APA	Lime N	Lime T
Sensitivity–coefficient of variation (%)			
Density	1.1	0.7	0.8
Depth of cone penetration	8.9	5.6	6.3
Flow diameter	4.2	1.8	3.1
Depth of plunger penetration	19.0	11.4	9.0
Air content *A_c_*	1.6	6.5	19.7
Compressive strength	44.5	32.4	26.6
Flexural strength	38.2	35.5	26.0

**Table 24:** The sensitivity of masonry mortar properties to temperature changes.

Property	APA	Lime N	Lime T
Sensitivity–coefficient of variation (%)			
Density	2.0	1.2	1.2
Depth of cone penetration	6.6	13.4	6.9
Flow diameter	6.5	4.1	2.0
Depth of plunger penetration	10.1	15.6	5.5
Air content *A_c_*	6.9	20.8	13.4
Compressive strength	52.0	22.3	20.7
Flexural strength	41.7	23.1	17.2

## References

[1] Panas J. (2010). Nowy Poradnik Majstra Budowlanego.

[2] Middendorf B., Hughes J.J., Callebaut K., Baronio G., Papayianni I. (2005). Investigative methods for the characterisation of historic mortars—Part 1: Mineralogical characterization. Mater. Struct..

[3] Groot C. (2012). RILEM TC 203-RHM: Repair mortars for historic masonry. Performance requirements for renders and plasters. Mater. Struct..

[4] Manita P., Triantafillou C. (2011). Influence of the design materials on the mechanical and physical properties of repair mortars of historic buildings. Mater. Struct..

[5] Raeis Samiei R., Daniotti B., Pelosato R., Dotelli G. (2015). Properties of cement–lime mortars vs. cement mortars containing recycled concrete aggregates. Constr. Build. Mater..

[6] Tan K.H., Du H. (2013). Use of waste glass as sand in mortar: Part I—Fresh, mechanical anddurability properties. Cem. Concr. Compos..

[7] Ortega J.M., Letelierb V., Solas C., Moriconi G., Climent M.Á., Sánchez I. (2018). Long-term effects of waste brick powder addition in the microstructure and service properties of mortars. Constr. Build. Mater..

[8] Gupta L.K., Vyas A.K. (2018). Impact on mechanical properties of cement sand mortar containing waste granite powder. Constr. Build. Mater..

[9] Buyuksagis I.S., Uygunoglu T., Tatar E. (2017). Investigation on the usage of waste marble powder in cement-based adhesive mortar. Constr. Build. Mater..

[10] Harbi R., Derabla R., Nafa Z. (2017). Improvement of the properties of a mortar with 5% of kaolin fillers in sand combined with metakaolin, brick waste and glass powder in cement. Constr. Build. Mater..

[11] Boukour S., Benmalek M.L. (2016). Performance evaluation of a resinous cement mortar modified with crushed clay brick and tire rubber aggregate. Constr. Build. Mater..

[12] Ge Z., Sun R., Zhang K., Gao Z., Li P. (2013). Physical and mechanical properties of mortar using waste Polyethylene Terephthalate bottles. Constr. Build. Mater..

[13] Rashid K., Ahmad M., Tahir M. (2018). Influence of organic agents to compressive strength of cement mortar. Constr. Build. Mater..

[14] Kim J., Yi C., Zi G. (2015). Waste glass sludge as a partial cement replacement in mortar. Constr. Build. Mater..

[15] Pliya P., Cree D. (2015). Limestone derived eggshell powder as a replacement in Portland cement mortar. Constr. Build. Mater..

[16] Jasiczak J., Zieliński K. (2006). Effect of protein additive on properties of mortar. Cem. Concr. Compos..

[17] Fang S., Zhang K., Zhang H., Zhang B. (2015). A study of traditional blood lime mortar for restoration of ancient buildings. Cem. Concr. Res..

[18] Zhang K., Rampazzi L., Riccardi M.P., Sansonetti A., Grimoldi A. (2018). Mortar mixes with oxblood: Historical background, model sample recipes and properties. Adv. Geosci..

[19] Zhao P., Xie D.Q., Li G.Y., Zhang Y.S. (2014). An Experimental Study of Pig Blood–Lime Mortar Used on Ancient Architecture in China. Adv. Mater. Res..

[20] Çomak B., Bideci A., Bideci Ö.S. (2018). Effects of hemp fibers on characteristics of cement based mortar. Constr. Build. Mater..

[21] Trejbal J. (2018). Mechanical properties of lime-based mortars reinforced with plasma treated glass fibers. Constr. Build. Mater..

[22] Schulze J. (1999). Influence of water-cement ratio and cement content on the properties of polymer-modified mortars. Cem. Concr. Res..

[23] Mahdi F., Ali Khan A., Abbas H. (2007). Physiochemical properties of polymer mortar composites using resins derived from post-consumer PET bottles. Cem. Concr. Compos..

[24] Keriene J., Antonovic V., Stonys R., Boris R. (2019). The influence of the ageing of calcium aluminate cement on the properties of mortar. Constr. Build. Mater..

[25] Reis J.M.L. (2012). Effect of Temperature on the Mechanical Properties of Polymer Mortars. Mat. Res..

[26] Sajedi F., Razak H.A. (2011). Effects of curing regimes and cement fineness on the compressive strength of ordinary Portland cement mortars. Constr. Build. Mater..

[27] Sajedi F. (2012). Effect of curing regime and temperature on the compressive strength of cement-slag mortars. Constr. Build. Mater..

[28] Garijo L., Xin Z.X., Ruiz G., Ortega J.J., Wu Z. (2018). The effects of dosage and production process on the mechanical and physical properties of natural hydraulic lime mortars. Constr. Build. Mater..

[29] Pavlík V., Uzáková M. (2016). Effect of curing conditions on the properties of lime, lime–metakaolin and lime–zeolite mortars. Constr. Build. Mater..

[30] Gołaszewski J. (2006). Wpływ Superplastyfikatorów na Właściwości Reologiczne Mieszanek na Spoiwach Cementowych w Układzie Zmiennych Czynników Technologicznych.

[31] Łukowski P. (2010). Kierunki rozwoju domieszek do betonu. Mat. Bud..

[32] Łukowski P. (2016). Domieszki do betonu—problem kompatybilności. Mat. Bud..

[33] Schuller M., Van der Hoeven R., Thomson M., Boyd J., Scheffler M. (1999). Comparative Investigation of Plastic Properties and Water Permeance of Cement-Lime Mortars and Cement-Lime Replacement Mortars. Water Problems in Building Exterior Walls: Evaluation, Prevention, and Repair.

[34] Lenart M. (2013). Impact Assessment of Lime Additive and Chemical Admixtures on Selected Properties of Mortars. Procedia Eng..

[35] Lawrence S.J., Cao H.T. An experimental study of the interface between brick and mortar. Proceedings of the 4th North Am. Mason. Conf..

[36] Groot C. (1995). Effects of water on mortar-brick bond. Heron.

[37] Kjaer E. (2010). Bond strength: What is influencing bond strength? In what way the bond strength influences mortar porperties. Proceedings of the 8th International Masonry Conference.

[38] O’Looney D., Pavía S. (2014). A Study of the Functionality of Hydrated Lime as an Admixture. J. Mater. Sci. Res..

[39] Souza A., De Carvalhais C.A., Dos Santos W.J. Comparative study between mortar with additive and mortar with hydraulic lime: Analysis of air entraining agent, water retainer and plasticizer. Proceedings of the VI Congresso de Engenharia Civil, Federal University of Juiz de Fora.

[40] Green K.M., Carter M.A., Hoff W.D., Wilson M.A. (1999). The effects of lime and admixtures on the water-retaining properties of cement mortars. Cem. Concr. Res..

[41] Wright B.T., Wilkins R.D., John G.W. (1993). Variables Affecting the Strength of Masonry Mortars.

[42] Gołaszewski J., Cygan G., Gołaszewska M. (2019). Effect of material and technological factors on the properties of cement-lime mortars and mortars with plasticizing admixture. Open Eng..

[43] Lanas J., Sirera R., Alvarez J.I. (2006). Study of the mechanical behavior of masonry repair lime-based mortars cured and exposed under different conditions. Cem. Concr. Res..

[44] Fusade L., Viles H.A. (2019). A comparison of standard and realistic curing conditions of natural hydraulic lime repointing mortar for damp masonry: Impact on laboratory evaluation. J. Cult. Herit..

[45] Mirza W.H., Al-Noury S.I., Al-Bedawi W.H. (1991). Temperature effect on strength of mortars and concrete containing blended cements. Cem. Concr. Compos..

[46] Soutsos M., Hatzitheodorou A., Kanavaris F., Kwasny J. (2017). Effect of temperature on the strength development of mortar mixes with GGBS and fly ash. Mag. Concr. Res..

[47] Korhonen C.J., Thomas R.D., Cortez E.R. Increasing Cold Weather Masonry Construction Productivity. https://rosap.ntl.bts.gov/view/dot/14086.

[48] Fishburn C.C. (1961). Effect of Mortar Properties on Strength of Masonry.

[49] Cavaco L., Veiga M.R., Gomes A., CIB LNEC Render Application Techniques for Ancient Buildings Técnicas de Aplicação de Argamassas de Revestimento em Edifícios Antigos. Proceedings of the 2nd International Symposium on Building Pathology, Durability and Rehabilitation.

[50] Rosell J.R., Laia H., Antonia N., Inma R. (2014). Cantalapiedra, Influence of the traditional slaking process on the lime putty characteristics. Constr. Build. Mater..

[51] Balksten K., Kenth K. The influence of craftsmanship on the inner structures of lime plasters. Proceedings of the International RILEM Workshop Repair Mortars for Historic Masonry.

[52] Sandin K. (1997). Mortars for Masonry and Rendering Choice and Application. Build. Issues.

[53] Davison J.I. (1976). The effect of mixing time on the compressive strength of masonry mortars. Build. Res. Note..

[54] Fukui E., Martins E.J., Campos H.F., Pinto M.C.C., Silva S.H.L.D., Rocha T.M.S., Kudlanvec J., Vitor L., Costa M.D.R.D.M. (2018). The influence of mixing procedure on fresh behavior of industrialized coating mortar and on site produced mortar. Matéria.

[55] (2016). CEN-EN 196-1:2016 Methods of Testing Cement—Part 1: Determination of Strength.

[56] (2000). PN-85-B-04500: Mortars. Physical and Mechanical Mests.

[57] (2000). PN-EN 1015-3:2000 Methods of Tests for Masonry—Part 3. Determination of Consistence of Fresh Mortar by Flow Table.

[58] (2000). PN-EN 1015-4:2000 Methods of Tests for Masonry—Part 4. Determination of Consistence of Fresh Mortar by Plunger Penetrator.

[59] (2000). PN-EN 1015-6:2000 Methods of Tests for Masonry—Part 6. Determination of Bulk Density of Fresh Mortar.

[60] (1998). PN-EN 1015-7:1998 Methods of Tests for Masonry—Part 7. Determination of Air Content of Fresh Mortar.

[61] (1999). CEN-EN 1015-11:1999 Methods of Tests for Mortar for Masonry—Part 11: Determination of Flexural and Compressive Strength of Hardened Mortar.

[62] Shang J., Yokota Y., Zhao Z., Dang W. (2018). DEM simulation of mortar-bolt interface behaviour subjected to shearing. Constr. Build. Mater..

[63] Aliyu M.M., Shang J., Murphy W., Lawrence J.A., Collier R., Kong F., Zhao Z. (2019). Assessing the uniaxial compressive strength of extremely hard cryptocrystalline flint. Int. J. Rock Mech. Min. Sci..

[64] Ferraris C.F. (1999). Measurement of the rheological properties of high performance concrete: State of the art report. J. Res. Natl. Inst. Stand. Technol..

[65] Struble L.J., Jiang Q. (2004). Effects of air entrainment on rheology. Aci Mater. J..

[66] Gao H., Zhang X., Zhang Y. (2015). Effect of the entrained air void on strength and interfacial transition zone of air-entrained mortar. J. Wuhan Univ. Technol. Sci. Ed..

[67] Peng Z., Dan L., Yun Q., Sulei Z., Congtao S., Tiejun Z. (2018). Effect of Air Entrainment on the Mechanical Properties, Chloride Migration, and Microstructure of Ordinary Concrete and Fly Ash Concrete. J. Mater. Civ. Eng..

[68] Zheng X., Li Q., Yuan J., Ge Y. (2012). The Flexural Strength and Frost Resistance of Air Entrained Concrete. Adv. Eng. Forum..

[69] Babiak M., Ratajczak M., Kulczewski P., Kosno J. (2018). Effect of modern air entraining admixtures on physical properties of construction mortars. Mater. Sci. Forum..

[70] Juvas K., Kaeppi A., Salo K., Nordenswan E. (2000). Effects of cement variations on concrete properties. Betonw. Und Fert. Precast. Plant Technol..

[71] Priyadarshana T., Dissanayake R. (2013). Importance of Consistent Cement Quality for a Sustainable Construction. Int. J. Mater. Mech. Manuf..

[72] Lyse I. (1935). Effect of brand and type of cement on strength and durability of concrete. J. Proc..

[73] Hendrickx R. (2009). The Adequate Measurement of the Workability of Masonry Mortar. Ph.D. Thesis.

[74] Casali J.M., Melo F.D., Serpa V.C., Oliveira A.L., De Betioli A.M., Calçada L.M.L. (2018). Influence of cement type and water content on the fresh state properties of ready mix mortar. Ambient. Construído..

[75] Elnemr A. (2019). Role of water-binder ratio on strength development of cement mortar AJER. J. Eng. Res..

[76] Lawrence R.M.H., Walker P. The impact of the water/lime ratio on the structural characteristics of air lime mortars, Structural Analysis of Historic Construction: Preserving Safety and Significance. Proceedings of the VI International Conference on Structural Analysis of Historic Construction, SAHC08.

[77] Kim Y.Y., Lee K.M., Bang J.W., Kwon S.J. (2014). Effect of W/C Ratio on Durability and Porosity in Cement Mortar with Constant Cement Amount. Adv. Mater. Sci. Eng..

[78] Qadir W., Ghafor K., Mohammed A. (2020). Evaluation the effect of lime on the plastic and hardened properties of cement mortar and quantified using Vipulanandan model. Open Eng..

[79] Han D., Ferron R.D. (2016). Influence of high mixing intensity on rheology, hydration, and microstructure of fresh state cement paste. Cem. Concr. Res..

[80] Cizer Ö., Rodriguez-Navarro C., Ruiz-Agudo E., Elsen J., Van Gemert D., Van Balen K. (2012). Phase and morphology evolution of calcium carbonate precipitated by carbonation of hydrated lime. J. Mater. Sci..

[81] Rostásy F.S., Schneider U., Wiedemann G. (1979). Behaviour of mortar and concrete at extremely low temperatures. Cem. Concr. Res..

[82] Krstulović P., Kamenić N., Popović K. (1994). A new approach in evaluation of filler effect in cement I. Effect on strength and workability of mortar and concrete. Cem. Concr. Res..

[83] Tynes W.O. (1968). Effect of Temperature on Air-Entraining Admixture Demand of Concrete with and Without Pozzolans.

[84] Silva W.R.L., Prudêncio L.R., Oliveira A.L., Damo G., Tochetto E. (2010). Influence of Air Temperature on the Performance of Different Water-Reducing Admixtures with Respect to the Properties of Fresh and Hardened Mortar. Adv. Civ. Eng..

[85] Gołaszewska M., Ponikiewski T. (2017). Influence of Low Pressure Steam Curing on Development of Strength of Mortars Based on Cement with High-calcium Fly Ash. Procedia Eng..

[86] Pimenta Teixeira K., Perdigão Rocha I., De Sá Carneiro L., Flores J., Dauer E.A., Ghahremaninezhad A. (2016). The Effect of Curing Temperature on the Properties of Cement Pastes Modified with TiO_2_ Nanoparticles. Materials.

